# Study on the Chemical Constituents and Anti-Migraine Activity of Supercritical CO_2_ Extracts of *Zanthoxylum schinifolium*

**DOI:** 10.3389/fphar.2021.744035

**Published:** 2021-11-30

**Authors:** Ruifang Yuan, Yunzhen Shi, Jinming Zhang, Qi Hu, Xichuan Wei, Chuanhong Luo, Yi Wu, Jinhui Yang, Ming Yang, Fang Wang, Chuan Zheng, Dingkun Zhang

**Affiliations:** ^1^ Pharmacy School, State Key Laboratory of Southwestern Chinese Medicine Resources, Chengdu University of Traditional Chinese Medicine, Chengdu, China; ^2^ Institute of Chinese Medical Sciences, State Key Laboratory Chuan of Quality Research in Chinese Medicine, University of Macau, Macau, China; ^3^ Sichuan Baicao Jinggong Biotechnology Co., Ltd., Chengdu, China; ^4^ Key Laboratory of Modern Preparation of Traditional Chinese Medicine Under Ministry of Education, Jiangxi University of Traditional Chinese Medicine, Nanchang, China; ^5^ Hospital of Chengdu University of Traditional Chinese Medicine, Chengdu, China

**Keywords:** *Zanthoxylum schinifolium*, supercritical CO_2_ extracts, migraine, nitroglycerin, reserpine, linalool

## Abstract

**Background:** *Zanthoxylum schinifolium* is a common herbal medicine in Southwest China. It is also a condiment commonly used in many families. In Chinese folk medicine, *Z. schinifolium* is considered to have the effect of relieving migraine, but there is no modern evidence on its anti-migraine mechanism.

**Objective:** The aim of this study was to investigate the chemical constituents of the supercritical carbon dioxide extracts of *Z. schinifolium* (CO_2_-ZSE) and its effects on migraine animals.

**Materials and Methods:** Supercritical CO_2_ extraction technology was applied to extract the dried fruit of *Z. schinifolium*, and the chemical components were determined by gas chromatography–mass spectrometry (GC-MS). Two migraine animal models were established by subcutaneous injection of nitroglycerin (NTG) and reserpine, respectively, to further evaluate the therapeutic effect of CO_2_-ZSE and explore its mechanism. On the basis of the experimental results, the therapeutic effects of linalool in different dosages and different ways of administration on NTG-induced migraine rats have been further investigated.

**Results:** About 125 peaks were detected in CO_2_-ZSE, and the relative content of linalool was 74.16%. CO_2_-ZSE decreased the number of head-scratching significantly and the levels of serum nitric oxide (NO), endothelin-1 (ET-1), calcitonin gene–related peptide (CGRP), interleukin-1β (IL-1β), nuclear factor kappa B (NF-κB) p65, and inhibitor of kappa B alpha (IκBα), and increased the level of 5-hydroxytryptamine (5-HT). Linalool has the potential to reduce the frequency of scratching the head and the expressions of NO, ET-1, and CGRP in NTG-induced migraine rats.

**Conclusion:** CO_2_-ZSE has a definite therapeutic effect on migraine by affecting the expression of vasomotor factors and the inflammatory pathway. Linalool has been proven to be the main effective substance against migraine. These findings provide scientific basis for the development of effective and simple migraine therapy.

## Introduction

Migraine is a common primary headache disease, characterized by unilateral or bilateral pulsatile pain, accompanied by photophobia, voice fear, nausea, vomiting, and other symptoms (Yuan et al., 2021). Many epidemiological studies have demonstrated its high prevalence and impact on social economy and individuals (Vos et al., 2017). The prevalence of migraine in China had reached 9.3% by 2012 (Sirri et al., 2018). Due to the recurrent attacks and difficulty in curing, it not only leads to serious physiological and psychological problems in patients but also places a huge economic burden on the family.

At present, there is no final consensus on the pathogenesis of migraine. More and more studies have shown that the migraine phase involves the activation of the trigeminocervical complex (TCC) (Goadsby, 2005), which triggers the inflammatory cascade and affects vasomotor function (Martins et al., 2017). In this pathophysiological process, some factors such as 5-HT (Deen et al., 2019), nitric oxide (NO), calcitonin gene–related peptide (CGRP), and endothelin-1 (ET-1) that affect vasomotor and the nuclear factor kappa B (NF-κB) inflammatory pathway play an important role (Iljazi et al., 2018). Triptan 5-hydroxytryptamine (5-HT) receptor agonists have been used in clinics as a migraine treatment drug, such as triptan drugs, but the safety of its long-term use remains to be investigated (de Vries et al., 2020). It is thus urgent to find a safe and effective anti-migraine drug.

**Graphical Abstract:**
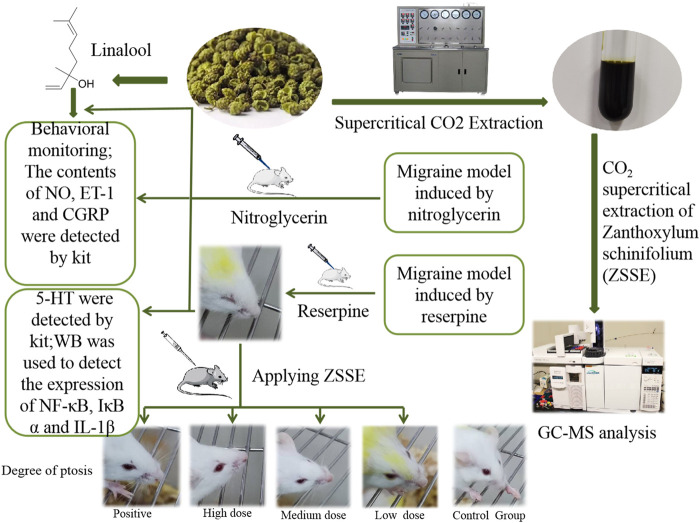


*Zanthoxylum schinifolium*, with strong aroma and hemp flavor, is the dry mature pericarp of *Zanthoxylum schinifolium* Sieb. et Zucc. (Meng et al., 2020). It is also a well-known condiment worldwide, especially in southern and northwestern China. The chemical constituents mainly include volatile oils, alkaloids, amides, coumarin, and lignin (Chen et al., 2016). Among them, amides are the main numb-taste components in *Z. schinifolium*, and volatile oil is the main aroma component. The pharmacological effects of *Z. schinifolium* are mainly focused on analgesic, anti-inflammatory, antibacterial, anticancer, antiviral properties, and so on (Tong and Wang, 1995; Cao et al., 2009). It was recorded in the book “*Properties Theory of Materia Medica* (yao xing lun)” in the Tang Dynasty that *Z. schinifolium* has the effect of treating migraine, and it is also used in the folk medicine. However, there is no evidence of classical model experiments on the treatment of migraine. Existing studies have shown that the volatile oil of *Z. schinifolium* has obvious analgesic effect, and the analgesic effect and duration of *Z. schinifolium* are stronger than those of *Zanthoxylum bungeanum* (Wu and Wu, 2014; Song and Liu, 2009). Therefore, the anti-migraine effect of *Z. schinifolium* was investigated *in vivo* in this study. The NTG and reserpine migraine models are recognized as reliable and practical and are widely used to study the mechanism of migraine. Accordingly, we studied the anti-migraine effect of the supercritical CO_2_ extracts of *Z. schinifolium* (CO_2_-ZSE), and explored the material basis of CO_2_-ZSE anti-migraine by discussing the dosage and different administration methods of linalool, the main component of CO_2_-ZSE. We believe that this research will provide a quick, simple, and effective relief method for migraine patients worldwide, and will attract more doctors and pharmaceutical workers to pay attention to the anti-migraine effect of *Z. schinifolium*.

## Materials and Methods

### Plant Material

The peel of dried *Z. schinifolium* was purchased from Sichuan Neautus Traditional Chinese Medicine Co., Ltd. (Chengdu, China) and authenticated by Xu Runchun (Associate Professor of Chengdu University of Traditional Chinese Medicine). A voucher specimen (ID 20191006) has been preserved in the School of Pharmacy, Chengdu University of Traditional Chinese Medicine.

### Equipment and Regents

The supercritical CO_2_ extraction device used to extract the volatile oil of *Z. schinifolium* was produced in Haian Huada Petroleum Instrument Co., Ltd. (Jiangsu, China). Nitroglycerin (NTG) injection was purchased from Beijing Yimin Pharmaceutical Co., Ltd. (Beijing, China). Reserpine was purchased from Chengdu Must Bio-technology Co., Ltd. (Chengdu, China). Zolmitriptan tablets were purchased from Venturepharm Pharmaceutical (Hainan) Co., Ltd. (Hainan, China). The commercial enzyme-linked immunosorbent assay (ELISA) kits for NO, 5-HT, CGRP, and ET-1 were purchased from Elabscience Biotechnology Co., Ltd. (Wuhan, China). IL-1β was purchased from Abcam (United Kingdom). NF-κB p65 was purchased from Immunoway (United States). GAPDH, BCA Protein Quantitative Detection Kit, HRP-labeled goat anti-rabbit, HRP-labeled goat anti-mouse, and HRP-labeled goat anti-rat were purchased from Multisciences (Lianke) Biotech Co., Ltd. (Hangzhou, China). IκBα, SDS-PAGE gel preparation kit, and phosphorylated protease inhibitor were purchased from Wuhan Servicebio Technology Co., Ltd. (Wuhan, China).

### Animals and Ethics Statements

Sprague–Dawley rats and Kunming mice used in this study were obtained from the Chengdu Dossy Experimental Animals Co., Ltd., and the animal license number was SCXK (Chuan) 2020-030. They were maintained at room temperature (25 ± 1°C) and humidity (60 ± 5%) with a 12-h light/dark cycle and free access to chow and water. This study was conducted in strict accordance with the recommendations of the Guidelines for the Care and Use of Laboratory Animals of the Ministry of Science and Technology of China. The protocol and experimental designs were approved by the Ethical Committee of Hospital of Chengdu University of Traditional Chinese Medicine (Approval ID: 2019KY-082).

### Preparation of CO_2_-ZSE

Two kilograms of *Z. schinifolium* was crushed by a pulverizer and separated by No. 10 screen, and the passing part was used to extract ZSE. The extraction pressure of the supercritical CO_2_ extraction device was 26 MPa, the temperature was 60°C, the analytical pressure was 7 MPa, the temperature was 55°C, the CO_2_ flow rate was 3 L·h^−1^, and the extraction time was 6 h. The calculation formula of the CO_2_-ZSE yield is given as follows:

$$CO_{2}-\mathrm{ZSE}\;yield/\%=\frac{M1/g}{M2/g}\times 100.$$ (1)

M1 represents the weight of CO_2_-ZSE and M2 represents the weight of *Z. schinifolium*.

### Gas Chromatography Mass Spectrometry Analysis of CO_2_-ZSE

1 ml of CO_2_-ZSE was put into a 10-ml volumetric flask, fixed with ethyl acetate, and then ultrasonically mixed. After 1 ml of the solution was taken out of the volumetric flask, it was filtered with a 0.45 μm microporous filter membrane, and the filtrate was used for GC-MS analysis.

The samples (1 µL each) were injected into the gas chromatograph system with a split inlet equipped with an HP-5 capillary column (30 m × 250 µm inner diameter and 0.25 µm film thickness) under the following conditions: initial oven temperature was set at 60 °C for 2 min, increased to 200°C at a rate of 5°C min^−1^ for 5 min, and then increased to 260°C at a rate of 10°C·min^−1^. Helium was applied as the carrier gas at a constant flow rate of 1 ml min^−1^, the injector temperature was set at 280°C, and the split ratio was 100:1.

The mass spectrometry conditions included a standard electron ionization (IE) source (70eV), an ion source temperature of 230°C, and an interface temperature of 150°C. The quadrupole mass analyzer had a scan range of 35–550 amu.

The components of CO_2_-ZSE were positively identified using the National Institute of Standards and Technology (NIST) 14.0 Mass Spectral Database. The semi-quantitative analysis of CO_2_-ZSE was performed by comparing their peak areas in the GC-MS total ion chromatogram. The percentage compositions of the compounds were calculated using the area normalization method.

### NTG-Induced Migraine Rats

Sixty adult Sprague–Dawley rats weighing 220–250 g, half male and half female, were randomly divided into six groups according to the weight, including control, model, positive zolmitriptan (0.25 mg·kg^−1^), high dose (250 mg·kg^−1^), medium dose (125 mg·kg^−1^), and low dose (62.5 mg·kg^−1^) of CO_2_-ZSE, respectively. On the day before the experiment, all the rats were depilated 2 cm × 3 cm on the back. The dosages of CO_2_-ZSE were determined according to the results of the preliminary experiments. Normal saline was applied on the back of the rats in the control group. NTG was injected subcutaneously into the rats (10 mg·kg^−1^) in other groups to copy the migraine models (Ni et al., 2019). CO_2_-ZSE was applied on the back of rats at 30 and 120 min after modeling. The positive group was administered zolmitriptan solution orally. The occurrence time of head scratching was recorded within 3 h after modeling, and the number of head scratching was recorded every 30 min. The occurrence time of head scratching was marked by more than five consecutive scratches. Four hours after the establishment of the migraine model, the rats in all groups were anesthetized by an intraperitoneal injection of 0.2 g·ml^−1^ urethane (1.5 g·kg^−1^). Subsequently, blood was taken from the abdominal aorta, and the brain tissue was rapidly excised to separate the TCC and then stored at −80°C until further processing.

### Reserpine-Induced Migraine Mice

Sixty Kunming mice weighing 18–22 g, half male and half female, were randomly divided into six groups according to body weight, which were control, model, positive zolmitriptan (0.36 mg·kg^−1^), high dose (360 mg·kg^−1^), medium dose (180 mg·kg^−1^), and low dose (90 mg·kg^−1^) of CO_2_-ZSE. On the day before the experiment, all the mice were depilated 2 cm × 3 cm on the back. Except the control group, the mice were injected subcutaneously with reserpine at a dose of 1.0 mg·kg^−1^ once a day for 10 days, with the mice in the control group receiving injections of isovolumic saline (Pu et al., 2019). After the establishment of the migraine model, RCXTA(in the CO2-ZSE groups), zolmitriptan (in the positive group), or tap water (in the control and model groups) were applied on the back of the mice for 5 days, One hour after the last administration, the eyeballs of the mice were taken and blood samples were collected. Then, the mice were euthanized, and the brain tissues were placed on ice and rapidly dissected while the isolated midbrain was immediately frozen and thereafter stored at −80°C until homogenization.

### Intervention Effect of Different Doses of Linalool on Nitroglycerin-Induced Migraine Rats

Thirty adult Sprague–Dawley rats weighing 220–250 g, half male and half female, were randomly divided into five groups according to body weight, including control, model, high dose (185 mg·kg^−1^), medium dose (92.5 mg·kg^−1^), and low dose (46.5 mg·kg^−1^) of linalool. The dosage of linalool was based on the amount of CO_2_-ZSE multiplied by the percentage of linalool. On the day before the experiment, all the rats were depilated 2 cm × 3 cm on the back. Except for the control group, NTG was injected subcutaneously into the rats (10 mg·kg^−1^) in each group to copy migraine models. Linalool was applied on the back of rats at 30 and 120 min after modeling, and normal saline was applied on the back of rats in the control group. The occurrence time of head scratching was recorded within 3 h after modeling, and the number of head scratching was recorded every 30 min. The occurrence time of head scratching was marked by more than five consecutive scratches.

### Intervention Effects of Different Administration Methods of Linalool on Nitroglycerin-Induced Migraine in Rats

Sixty adult Sprague–Dawley rats weighting 220–250 g, half male and half female, were randomly divided into six groups according to body weight, which were the normal, model, positive, linalool oral administration, linalool transdermal administration, and linalool inhalation groups. On the day before the experiment, all the rats were depilated 2 cm × 3 cm on the back. The migraine model was copied by subcutaneous injection of 10 mg·kg^−1^ NTG into the back of the neck of each group. The positive group was given 0.25 mg·kg^−1^ zolmitriptan, the control group was given the same amount of normal saline, and the linalool oral administration group was given linalool by gavage at 30 and 120 min. Linalool was applied on the back of the rats in the linalool transdermal administration group at the same time, and the rats in the linalool inhalation group were placed in the sniffing device; the dosage of linalool was 185 mg·kg^−1^. The occurrence time of head scratching was recorded within 3 h after modeling, and the number of head scratching was recorded every 30 min. The occurrence time of head scratching was marked by more than five consecutive scratches. Four hours after the establishment of the migraine model, the rats in all groups were anesthetized by an intraperitoneal injection of 0.2 g·ml^−1^ urethane (1.5 g·kg^−1^). Subsequently, blood was taken from the abdominal aorta until further processing.

### Enzyme-Linked Immunosorbent Assay

The levels of 5-HT in the brainstem tissue and NO, CGRP, and ET-1 in serum were determined by ELISA. The tissue was mixed with nine times the homogenate medium and ground. Then the ground solution was centrifuged at 3,000–4,000 r·min^−1^ for 10 min. The supernatant was prepared into 10% tissue homogenate and used immediately or directly frozen, and stored at −80 C. The serum was separated by centrifugation at 3,000 r·min^−1^ for 10 min at 4°C. Each multiplex assay was performed in accordance with the manufacturer’s instructions. The absorbance was measured in the microplate spectrophotometer.

### Western Blotting Analysis

The tissue was thoroughly homogenized with 10 times the tissue volume of the RIPA lysate on ice and centrifuged at 4°C at 12000 r·min^−1^ for 10 min, and the supernatant was separated and collected. The total protein was extracted according to the manufacturer’s instructions, and the protein concentration was determined by the BCA method. The protein was separated by SDS-PAGE and transferred onto the PVDF membrane, and the transformed membrane was placed on a decolorizing shaker at room temperature and sealed with 5% skimmed milk (0.5%TBST) for 1 h. The diluted primary antibody (5% skim milk dissolved by TBST, phosphorylated protein using 5% BSA dissolved by TBST) was incubated overnight at 4°C, and then incubated with the secondary antibody at room temperature for 30 min. ChemiScope Capture software filmed and quantified the samples. The intensity of the GAPDH protein band was used as the internal control.

### Statistical Analysis

The experimental data were expressed as mean ± standard error of the mean (SEM) (‾x ± s). The experimental data were statistically analyzed and plotted with SPSS 21.0. One-way analysis of variance (ANOVA) was used for comparison between groups. A *p* value < 0.05 indicates a significant difference, and a *p* value < 0.01 indicates a very significant difference.

## Results

### CO_2_-ZSE Yield

The results showed that the extraction rate of ZSE by supercritical CO_2_ extraction was 12.3%.

### GC-MS Analysis of CO_2_-ZSE

**FIGURE 1:**
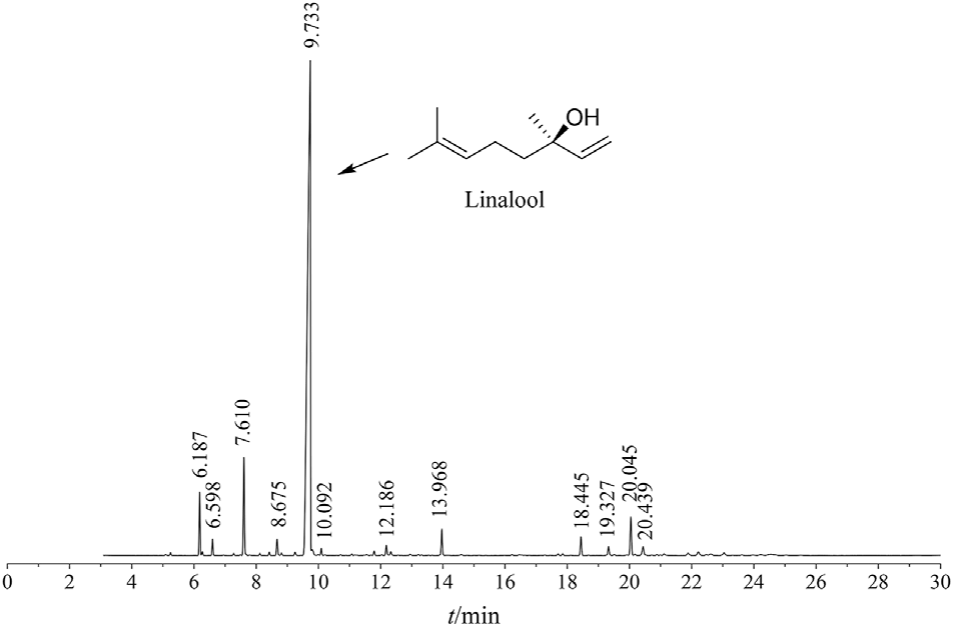
Total ion flow map of ZSSE

The ZSE extracted by supercritical CO_2_ extraction showed a total of 125 peaks by GC-MS analysis. Thirty-seven chemical constituents were identified. As shown in Figure 1 and Table 1, the main component of CO_2_-ZSE is linalool, accounting for 74.16% of the total volatile oil, followed by D-limonene and sabinene, with contents of 5.45 and 3.19%, respectively.

**TABLE 1:** Composition and relative content of ZSSE.

Peak number	Retention time/min	Chemical compound	Molecular formula	CAS number	Relative content/%
1	5.087	Alpha-thujene	C_10_H_16_	002867-05-2	0.06
2	5.251	Alpha-pinene	C_10_H_16_	000080-56-8	0.14
3	6.187	Sabinene	C_10_H_16_	003387-41-5	3.19
4	6.269	Beta-pinene	C_10_H_16_	000127-91-3	0.2
5	6.598	Beta-myrcene	C_10_H_16_	000123-35-3	0.82
6	6.957	Alpha-phellandrene	C_10_H_16_	000099-83-2	0.03
7	7.281	2-carene	C_10_H_16_	1000149-94-6	0.11
8	7.498	*p*-cymene	C_10_H_14_	000099-87-6	0.04
9	7.610	D-limonene	C_10_H_16_	005989-27-5	5.45
10	8.122	Beta-ocimene	C_10_H_16_	013877-91-3	0.11
11	8.422	Gamma-terpinene	C_10_H_16_	000099-85-4	0.18
12	8.675	*cis*-beta-terpineol	C_10_H_18_O	007299-40-3	1.04
13	8.816	*cis*-alpha,alpha,5-trimethyl-5-vinyltetrahydrofuran-2-methanol	C_10_H_18_O_2_	005989-33-3	0.15
14	9.733	Linalool	C_10_H_18_O	000078-70-6	74.16
15	9.998	Phenylethyl alcohol	C_8_H_10_O	000060-12-8	0.07
16	10.092	Thujone	C_10_H_16_O	000546-80-5	0.4
17	11.075	(+)-Citronellal	C_10_H_18_O	002385-77-5	0.11
18	11.798	(-)-4-terpineneol	C_10_H_18_O	020126-76-5	0.27
19	12.945	Gamma-terpinene	C_10_H_16_	000099-85-4	0.11
20	13.545	Cuminaldehyde	C_10_H_12_O	000122-03-2	0.03
21	13.663	(-)-carvone	C_10_H_14_O	006485-40-1	0.02
22	13.904	Cyclofeuchene	C_10_H_16_	000488-97-1	0.02
23	13.968	Linalyl acetate	C_12_H_20_O_2_	000115-95-7	1.54
24	16.227	5,9,9-trimethyl-spiro[3.5]non-5-en-1-one	C_12_H_18_O	1000185-13-4	0.06
25	17.856	Tetradecane	C_14_H_30_	000629-59-4	0.12
26	18.445	Caryophyllene	C_15_H_24_	000087-44-5	1.21
27	18.686	Cubebene	C_15_H_24_	013744-15-5	0.03
28	19.327	Humulene	C_15_H_24_	006753-98-6	0.66
29	20.045	Germacrene D	C_15_H_24_	023986-74-5	2.71
30	20.18	γ-Selinene	C_15_H_24_	000515-17-3	0.11
31	20.439	Bicyclogermacrene	C_15_H_24_	067650-90-2	0.85
32	20.88	Calarene	C_15_H_24_	017334-55-3	0.08
33	21.115	Delta-cadinene	C_15_H_24_	000483-76-1	0.17
34	21.886	Elemol	C_15_H_26_O	000639-99-6	0.27
35	22.215	Nerolidol	C_15_H_26_O	040716-66-3	0.42
36	22.474	(Z)-3-hexadecene	C_16_H_32_	034303-81-6	0.1
37	23.038	Hexadecane	C_16_H_34_	000544-76-3	0.31
		Total percentage of identified compounds			95.35

### Effect of CO_2_-ZSE on Nitroglycerin-Induced Migraine Rats

#### Behavioral Investigation

**FIGURE 2:**
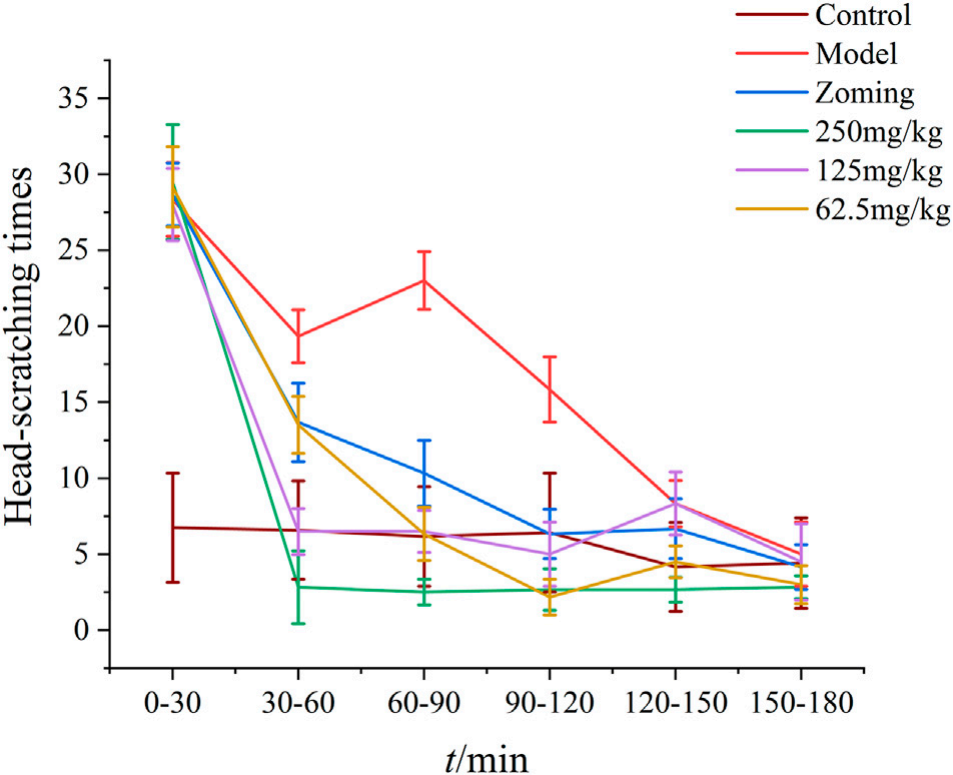
Effect of ZSSE on scratching times of migraine rats induced by NTG (‾x±SD, *n* = 10)

After subcutaneous injection of NTG for 3–5 min, red ears, frequent head scratching, cage climbing, and photophobia began to appear in rats, which lasted for at least 3 h and then gradually disappeared, and the rats entered a quiet state. As shown in Figure 2, the control rats occasionally scratched their heads and climbed into the cage within 3 h of behavioral monitoring. After treatment with the drugs, the number of head scratching was reduced in different degrees in the zolmitriptan group, and all doses of CO_2_-ZSE groups (*p-value* < 0.05, *p-value* < 0.01). The effect of high dose and low dose in the CO_2_-ZSE groups lasted for at least 150 min.

#### Determination of NO, CGRP, and ET-1 in Serum

**FIGURE 3:**
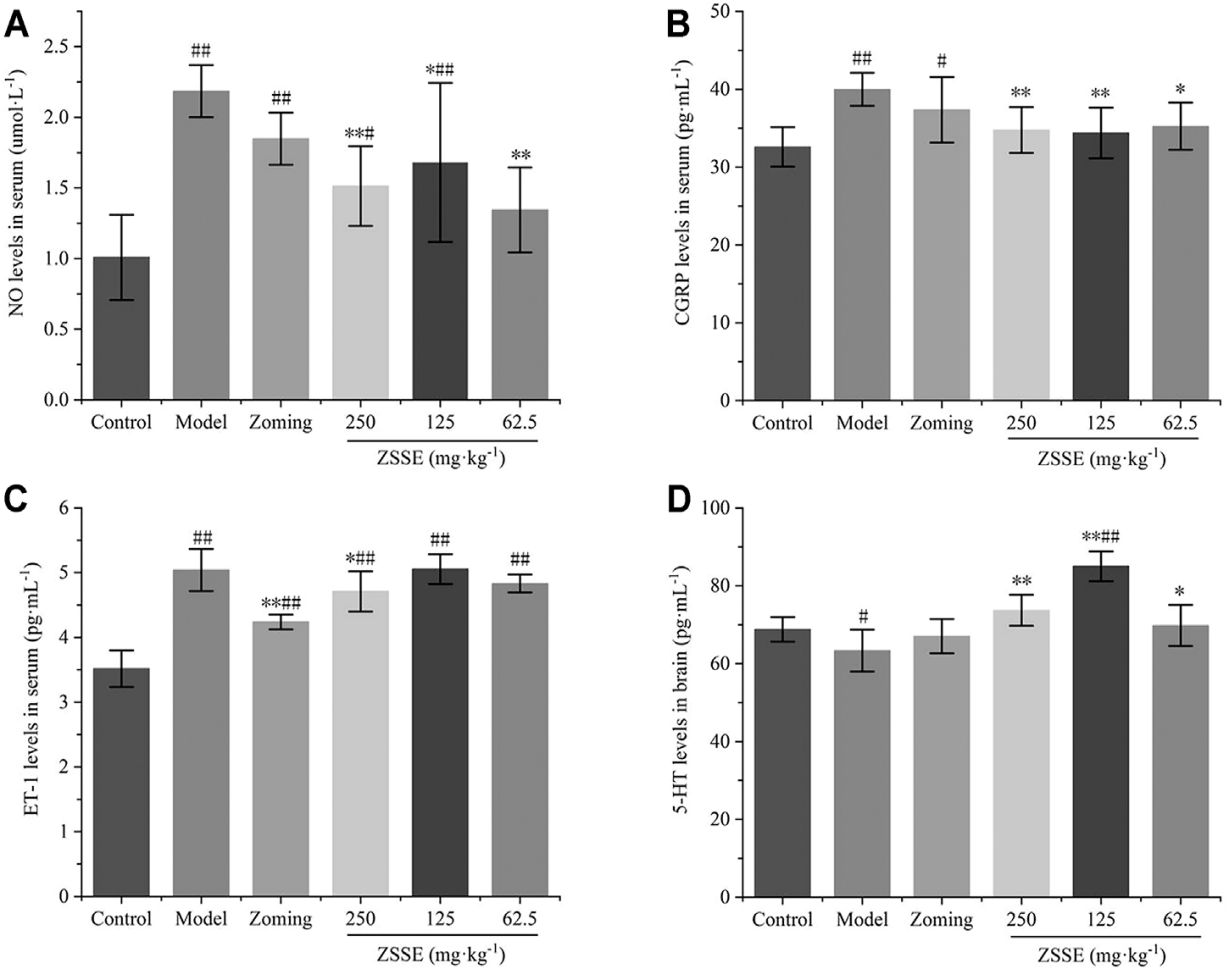
Effect of ZSSE on serum NO, CGRP, and ET-1 levels and TCC 5-HT levels in NTG-induced migraine rats (‾x±SD, *n* = 10). **(A)** NO levels in serum; **(B)** CGRP levels in serum; **(C)** ET-1 levels in serum; **(D)** 5-HT levels in the TCC. ^#^ *p* < 0.05, ^##^ *p* < 0.01 vs the control group, ^*^ *p* < 0.05, ^**^ *p* < 0.01 vs the model group.

The contents of NO, CGRP, and ET-1 in rat serum are shown in Figure 3. Compared with the control group, subcutaneous injection of NTG significantly increased the contents of NO, CGRP, and ET-1 in the serum of the model group (*p-value* < 0.01). Zolmitriptan decreased the expression of these three factors to some extent. High dose of CO_2_-ZSE significantly decreased the expression of NO, CGRP, and ET-1, while medium dose of CO_2_-ZSE significantly downregulated the expression of NO and CGRP, but had no significant effect on the content of ET-1. Low dose of CO_2_-ZSE significantly decreased the expression of NO and CGRP (*p* < 0.05, *p* < 0.01), and inhibited the expression of ET-1 with no significance.

#### Determination of the 5-HT Level in the Trigemino-Cervical Complex

The results of Figure 3 showed that NTG could significantly reduce the expression of 5-HT in the TCC of rats, zolmitriptan could upregulate the expression of 5-HT, 250 mg·kg^−1^ and 62.5 mg·kg^−1^ of CO_2_-ZSE could significantly increase the expression of 5-HT, and the upregulation effect of 125 mg·kg^−1^ of CO_2_-ZSE on 5-HT was the most obvious, which was significantly higher than that in the TCC of control rats.

#### Determination of Interleukin-1β, NF-κBp65, and Inhibitor of kappa B Alpha in the TCC

**FIGURE 4:**
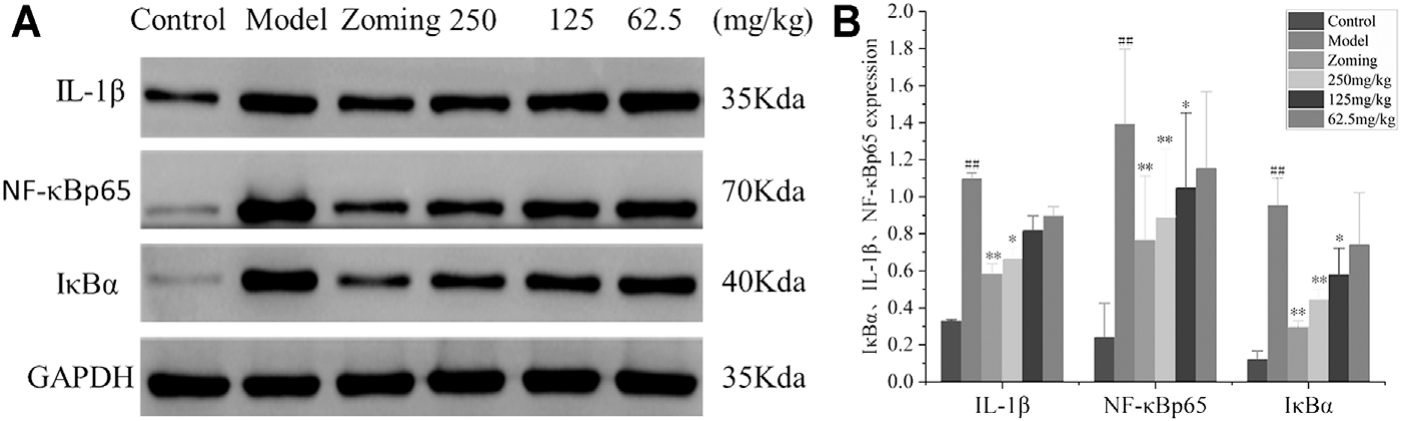
Western blotting analysis of IκBα, IL-1β, and NF-κBp65 expressions in the TCC of NTG-induced migraine rats (‾x±SD, *n* = 4). **(A)** WB strips; **(B)** Grayscale scan results of IL-1β, NF-κBp65, and IκBα. ^#^ *p* < 0.05, ^##^ *p* < 0.01 vs the control group, ^*^ *p* < 0.05, ^**^ *p* < 0.01 vs the model group.

The expressions of IL-1β, NF-κBp65, and IκBα in the TCC are shown in Figure 4. Compared with the expressions of IL-1β, NF-κBp65, and IκBα in the TCC site of the control group, the levels of IL-1β, NF-κBp65, and IκBα in the model group treated with subcutaneous injection of NTG were significantly increased (*p* < 0.01). The rats injected with NTG were treated with zolmitriptan and CO_2_-ZSE. It was found that zolmitriptan and a high dose of ZSE could significantly reverse the upregulation of IL-1β, NF-κBp65, and IκBα induced by NTG (*p-value* < 0.05). The medium dose of CO_2_-ZSE could significantly reduce the expression of NF-κBp65 and IκBα, and also inhibit the expression of IL-1β, but not significantly. Low dose of CO_2_-ZSE could reduce the expression of these three factors. However, there was no significant difference compared with the model group.

### Effect of CO_2_-ZSE on Reserpine Migraine Model Mice

#### Behavioral Investigation

Three days after subcutaneous injection of reserpine, the mice showed lethargy, the eyes narrowed slightly, curled up and showed little activities, and body temperature decreased. With the increase of modeling time, to the 10^th^ day of modeling, the eyes of mice had narrowed into a linear shape, and the body temperature was significantly lower than that of the normal group. The phenomenon of limb tremor occurred occasionally, which was consistent with the description in the literature (Lou et al., 2012).

#### Determination of NO, CGRP, and ET-1 Levels in Serum

**FIGURE 5:**
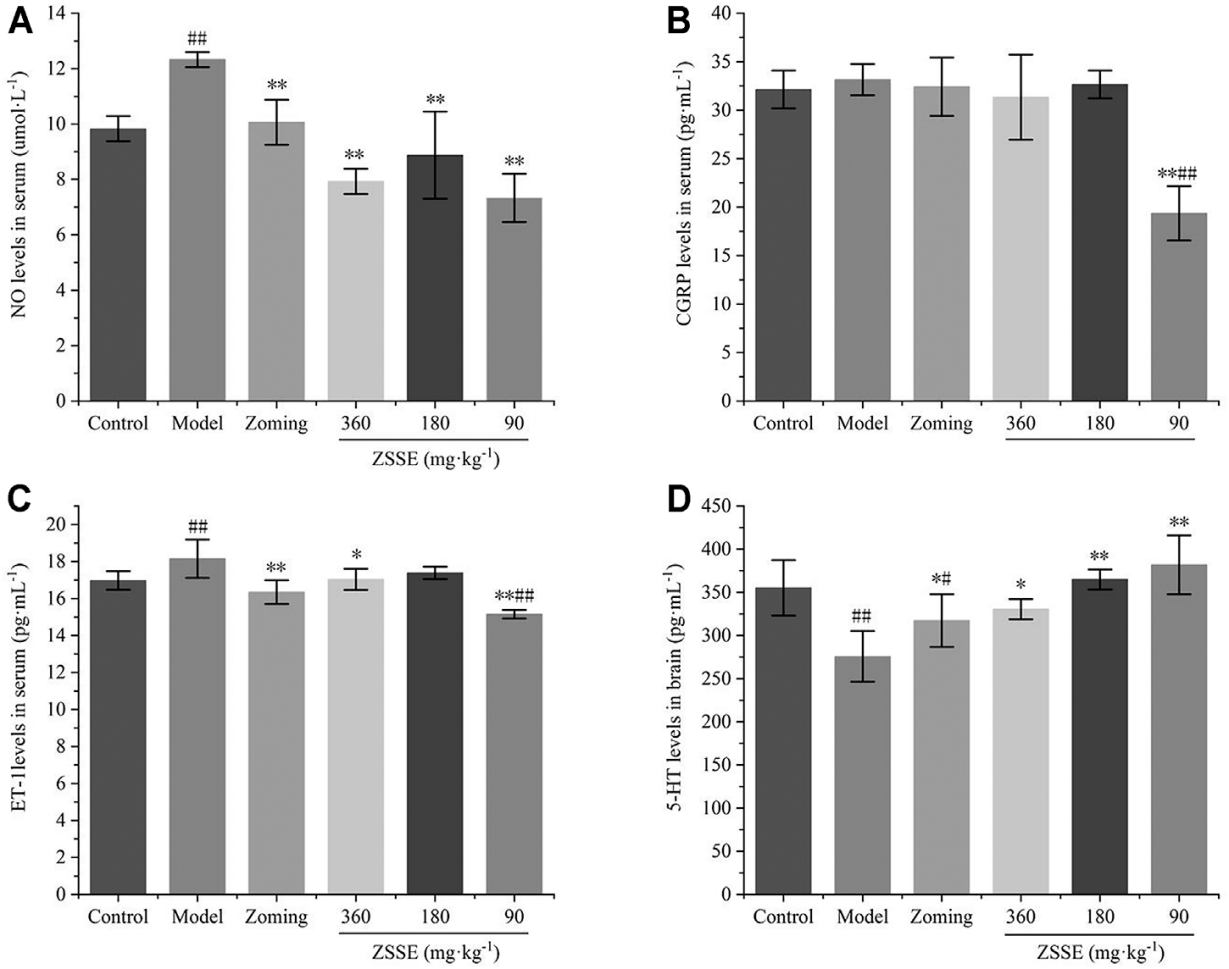
Effects of ZSSE on the levels of NO, CGRP, and ET-1 in serum and 5-HT in the brain tissue of migraine rats with reserpine (‾x±SD, *n* = 10). **(A)** NO levels in serum; **(B)** CGRP levels in serum; **(C)** ET-1 levels in serum; **(D)** 5-HT levels in the TCC. ^#^ *p* < 0.05, ^##^ *p* < 0.01 vs the control group, ^*^ *p* < 0.05, ^**^ *p* < 0.01 vs the model group.

The determination of NO, CGRP, and ET-1 levels is shown in Figure 5. Compared with the control group, reserpine could significantly increase the contents of NO and ET-1 in the serum of the model group, and had an increasing trend on the content of CGRP, but it was not significant. Zolmitriptan and high and low dose of CO_2_-ZSE could significantly reduce the expression of NO and ET-1 (*p* < 0.05, *p* < 0.01). Zolmitriptan and high and low dose of CO_2_-ZSE could significantly reduce the expression of NO and ET-1 (*p* < 0.05, *p* < 0.01), Medium dose of CO_2_-ZSE could significantly reduce the expression of NO. It is worth mentioning that zolmitriptan and high and medium doses of CO_2_-ZSE can downregulate the expression of CGRP, but not significantly. Low doses of CO_2_-ZSE can significantly downregulate the expression of CGRP and ET-1, which is significantly lower than that of the control group.

#### Determination of 5-HT Level in Brain

The 5-HT level in the brain is shown in Figure 5. Reserpine significantly decreased the expression of 5-HT in the brain, zolmitriptan could upregulate the expression of 5-HT, and high, medium, and low dose of CO_2_-ZSE could significantly increase the expression of 5-HT (*p* < 0.05, *p* < 0.01). The upregulation effect of low dose of CO_2_-ZSE on 5-HT was the most obvious, which was higher than that in the brain of control rats, but there was no significant difference.

#### Determination of IL-1β, NF-κBp65, and I κ B α Levels in the Brain

**FIGURE 6:**
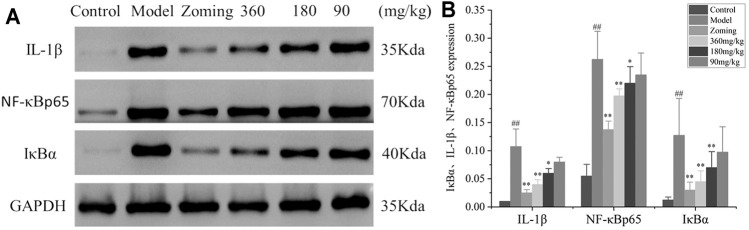
Western blot analysis of I κ B α, IL-1β, and NF-κbp65 expressions in the brain tissue of reserpine-type migraine rats (‾x±SD, *n* = 4). **(A)** WB strips; **(B)** Grayscale scan results of IL-1β, NF-κBp65, and IκBα. ^#^ *p* < 0.05, ^##^ *p* < 0.01 vs the control group, ^*^ *p* < 0.05, ^**^ *p* < 0.01 vs the model group.

The determination of IL-1β, NF-κBp65, and IκBα levels is shown in Figure 6. Compared with the expressions of IL-1β, NF-κBp65, and IκBα in the brain of the control group, the expressions of IL-1β, NF-κBp65, and IκBα were significantly increased in the model group treated with subcutaneous injection of NTG. The rats injected with NTG were treated with zolmitriptan and CO_2_-ZSE. It was found that zolmitriptan and high and medium doses of CO_2_-ZSE could significantly reverse the upregulation of IL-1β, NF-κBp65, and IκBα induced by nitroglycerin (*p* < 0.05, *p* < 0.05). Low dose of CO_2_-ZSE could significantly reduce the expression of IL-1β, NF-κBp65, and IκBα, but the effect is not obvious.

### Intervention Effect of Different Doses of Linalool on Nitroglycerin Migraine Rats

**FIGURE 7:**
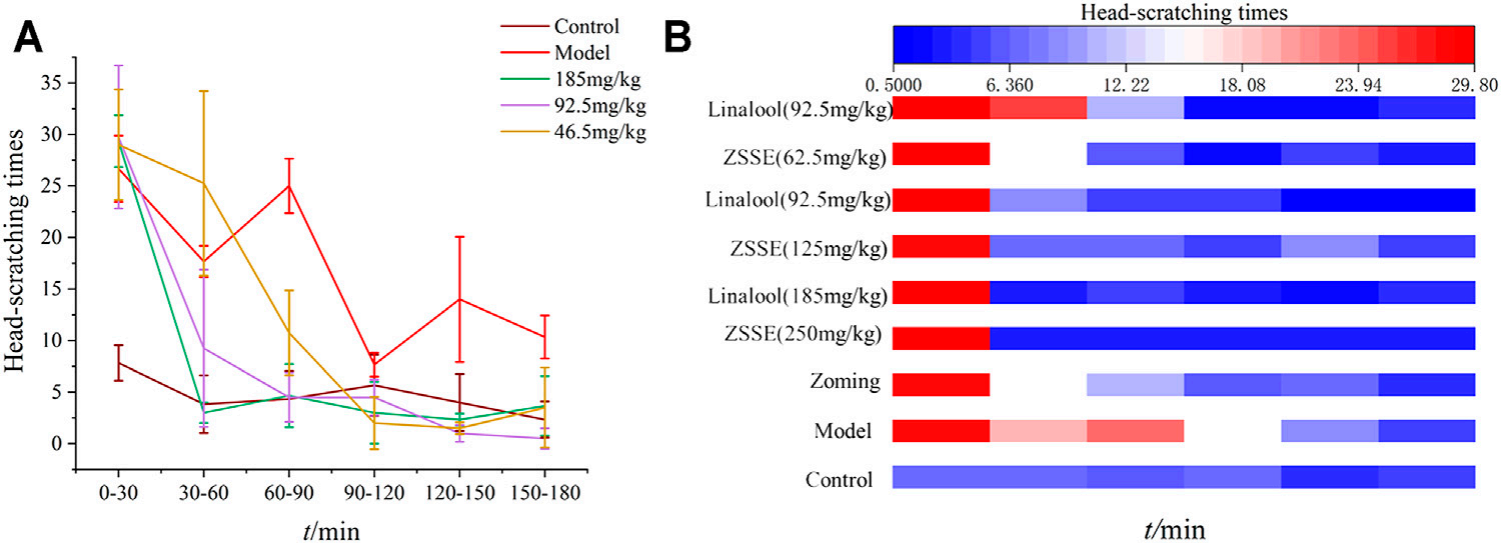
Number of head scratching in rats (‾x±SD, *n* = 6). **(A)** Effect of linalool intervention on scratching times of migraine rats induced by NTG; **(B)** comparison of the effects of ZSSE and linalool on the number of head scratching in rats.

After the rats were injected with NTG, the behavioral characteristics were consistent with those described in “3.3.1 behavioral investment.” The rats in the normal group had no red ear symptoms and occasionally scratched their heads to climb the cage within 3 h of behavioral monitoring. As shown in Figure 7, after the intervention of linalool, the high and medium doses of linalool could significantly reduce the number of head scratching in rats within 30 min, and this effect lasted at least 150 min, especially in the high-dose group. The number of head scratching in the low-dose linalool group suddenly increased 30 min after administration, even higher than that in the model group, and decreased slowly after 60 min of administration, indicating that different doses of linalool may have different regulatory effects on NTG-induced migraine rats. Low dose of linalool aggravates migraine symptoms, while high dose of linalool can relieve migraine symptoms. In addition, the effects of linalool and CO_2_-ZSE with the same amount of linalool on the number of head scratching in migraine rat models induced by NTG were basically the same, indicating that linalool is, indeed, the main active component of CO_2_-ZSE against migraine.

### Intervention Effects of Different Administration Methods of Linalool on Nitroglycerin-Induced Migraine in Rats

#### Behavioral Investigation

**FIGURE 8:**
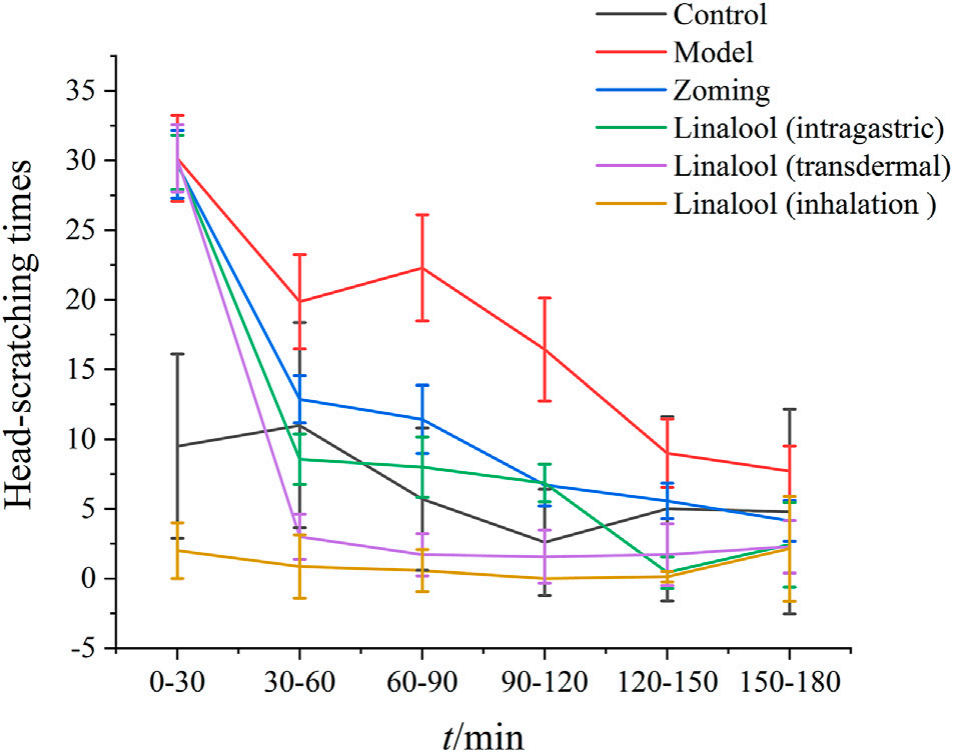
Number of head scratching in rats (‾x±SD, *n* = 10)

According to the therapeutic effect, the dosage of linalool 185 mg·kg^−1^ each time was selected in this experiment. As shown in Figure 8, there were significant differences in the effects of different administration methods of linalool on the number of head scratching in rats with NTG-induced migraine. Within 0–30 min, compared with the normal group, NTG significantly increased the number of head scratching in the other five groups, indicating that the model was successfully established. After 30 min of linalool administration, the number of head scratching decreased significantly in the oral administration and transdermal administration groups, especially in the transdermal administration group, and the effect lasted at least 150 min. The head scratching behavior of the rats in the olfactory administration group was always in a state of little activity, and there were significant differences among the rats. In the course of the experiment, the rats were basically in a state of eye-closing and motionless. It may be mainly because the relatively airtight space of the sniffing device affects the behavior of the rats, so the behavioral characteristics of the rats in this group cannot be used as a standard to evaluate the therapeutic effect, and the best mode of administration can be determined by further examination of rat serum factors.

#### Determination of NO, CGRP, and ET-1 in Serum

**FIGURE 9:**
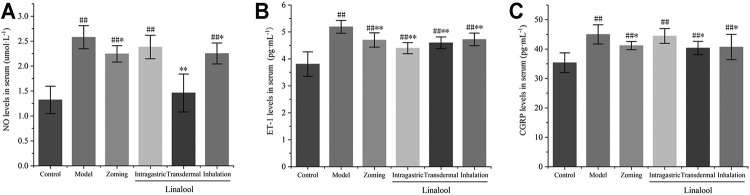
Effects of different administration methods of linalool on serum NO, ET-1, and CGRP levels in migraine rats (‾x±SD, *n* = 10). **(A)** NO levels in serum; **(B)** ET-1 levels in serum; **(C)** CGRP levels in serum. ^#^ *p* < 0.05, ^##^ *p* < 0.01 vs the control group, ^*^ *p* < 0.05, ^**^ *p* < 0.01 vs the model group.

As shown in Figure 9, compared with the control group, NTG could significantly increase the contents of NO, ET-1, and CGRP in the serum of the model group. Zolmitriptan can reduce the expression of these three factors to some extent. Oral administration of linalool significantly decreased the expression of ET-1, but had no significant effect on the contents of NO and CGRP. Transdermal administration of linalool could significantly downregulate the expressions of NO, ET-1, and CGRP (*p* < 0.05). Inhaling linalool could also significantly reduce the expressions of NO, ET-1, and CGRP (*p* < 0.05, *p* < 0.01), but the inhibitory effect on the expression of NO was not as significant as that of transdermal administration. The aforementioned results show that among the three modes of linalool administration, the effect of transdermal administration is the most significant, sniffing administration is the second, and oral administration is the worst. The shortcomings of oral administration, such as first-pass effect and slow absorption, may be the main reasons for the insignificant effect of linalool.

## Discussion

Migraine, as the second leading cause of disability in the world (Vos et al., 2017), is related to the health and quality of life of most people around the world. Current research shows that NO, 5-HT, CGRP, and NF-κB pathways play an important role in the pathophysiology of migraine. NO is a key molecule in the pathogenesis of migraine (Olesen, 2008). Excessive production of NO in the vascular smooth muscle cells will lead to excessive vasodilation, which, in turn, activates the nociceptive nerve fibers in the vascular wall and mediates the release of vasoactive substances such as CGRP, which further triggers perivascular neurogenic inflammation to participate in the occurrence of migraine (Yao et al., 2020). The pathophysiological changes of migraine rats induced by this model are also similar to those of human migraine. Reserpine is a monoamine neurotransmitter depletion agent that can reduce 5-HT in the body and cause migraine (Pu et al., 2019).

5-HT is recognized as a neurotransmitter closely related to migraine (Vila-Pueyo, 2018). During the aura of migraine attack, the increase of the 5-HT level causes vasoconstriction, which is then metabolized into 5-HIAA excreted in urine. Low concentration of 5-HT stimulates perivascular pain fibers, and local release of NO, prostaglandins (PGs), and neuropeptides cause vasodilation and enter the migraine attack.

CGRP is a kind of vasoactive neuropeptide composed of amino acids, which has the same vasodilative capacity as that of NO (Sohn et al., 2020). ET-1 is an endogenous vasoconstrictor released by the vascular endothelium (Hougaard et al., 2020), and it is also the strongest vasoconstrictor known at present.

The NF-κB pathway plays an important role in neurogenic inflammation of migraine (Antonova et al., 2013). Under normal physiological conditions, the phosphorylation site of NF-κB is blocked by IκB. After inflammatory injury, IKK kinase degrades IκB, exposing the nuclear localization signal of p50 and rapidly transferring p65 to the nucleus. p65 can bind to the κ B sites of some inflammatory factor gene promoters or enhancers by identifying specific DNA sequences, which starts the transcription of related genes and induces the overexpression of a variety of cytokines to cause inflammation (Reuter et al., 2002). Previous studies have shown that inflammatory factors such as TNF-α, IL-1β, and IL-6 can be induced by NF-κB (Li et al., 2019).

In this experiment, the rat model of NTG-induced migraine and the mouse model of reserpine migraine with good repeatability, economy, and simplicity were selected to explore the therapeutic effect of CO_2_-ZSE on migraine. NTG and reserpine can regulate vascular tension and neurotransmitter balance by regulating vasomotor factors such as NO, CGRP, and ET-1 and neurotransmitters such as 5-HT, resulting in the activation and accumulation of proinflammatory mediators, finally leading to migraine (Sun et al., 2017; Wang et al., 2019). We found that CO_2_-ZSE decreased the number of head scratching significantly; the levels of NO, ET-1, and CGRP; and the levels of IL-1β, NF-κB p65, and inhibitor of IκBα, and increased the level of 5-HT in NTG-induced rats or reserpine-induced mice. This indicates that CO_2_-ZSE reverted both migraine models, and it mainly regulated the upstream inflammatory pathway by affecting vasomotor and neurotransmitter levels to achieve therapeutic effect.

The content of linalool in CO_2_-ZSE is 74.16%. It is doubtful whether linalool can play an anti-migraine effect. At present, studies have shown that linalool has significant neurointervention and anti-inflammatory and analgesic effects, and can relieve anxiety (Harada et al., 2018) and depression (Goadsby, 2005) and cause a certain neuroprotective effect. This antidepressant effect is reflected by the interaction with the monoaminergic pathway. Linalool can also block the phosphorylation of the IkBa protein in raw 264.7 cells stimulated by lipopolysaccharide (LPS) and inhibit the NF-κB pathway (Huo et al., 2013), and the expression of inflammatory factors such as IL-1β, NO, and TNF-α, which reflects the anti-inflammatory effect (Batista et al., 2010; Li et al., 2015). Linalool can also reduce mechanical pain and hypersensitivity in neuropathic animals (Batista et al., 2010; Berliocchi et al., 2009). Some studies speculate that linalool has anti-migraine effects (Prachayasittikul et al., 2018; Delavar Kasmaei et al., 2016). Based on the aforementioned studies, we studied the effects of linalool on the behavior of NTG-induced migraine rats and found that applying the same amount of linalool to CO_2_-ZSE could reduce the number of head scratching in rats to the same extent, that is, linalool may be the main active component of CO_2_-ZSE against migraine. Using the same animal model, the therapeutic effects of oral administration, transdermal administration, and inhalation administration of linalool were compared by detecting the animal behavior and the contents of serum NO, ET-1, and CGRP. It was determined that transdermal administration of linalool was the preferred mode of administration because it significantly restored the levels of NO, CGRP, and ET-1 in the serum of migraine-induced mice to normal, followed by inhalation administration, and oral administration was the worst. This study indicates that linalool can also regulate the activation of upstream inflammatory pathways and the accumulation of inflammatory mediators by regulating vasomotor retraction, and finally play an anti-migraine role. This mechanism is similar to the main mechanism of other known effective molecules, such as parthenolide (Tassorelli et al., 2005).

At present, oral administration, acupuncture, massage, and aromatherapy are mostly used in the treatment of migraine, or they are used together (Yuan et al., 2021). There are few therapeutic drugs for percutaneous and sniffing administration, which limited the choice of medication for migraine patients. Our research has proved that linalool can play a good therapeutic effect on migraine through percutaneous administration, which will provide guiding significance for the research and development of migraine drugs in the future. However, we only studied the effect of linalool on migraine caused by the dysregulation of the inflammatory pathway. Indeed, the etiology of migraine is complex. We can study the therapeutic effect of linalool on other migraine models and expand the therapeutic range of linalool.

## Conclusion

CO_2_-ZSE can effectively treat migraine, increase the expression of 5-HT, decrease the expression of NO, CGRP, and ET-1 to regulate vasomotor and inhibit the expression of NF-κBp65, IκBα, and IL-1β to alleviate neurogenic inflammation. This effect mainly contributes to the high concentration of linalool. These findings reveal the scientific connotation, mechanism of action, and material basis of *Z. schinifolium* for the treatment of migraine.

## Data Availability

The raw data supporting the conclusions of this article will be made available by the authors, without undue reservation.

## Abbreviations

CO_2_-ZSE, supercritical CO_2_ extraction of *Zanthoxylum schinifolium*; CGRP, calcitonin gene–related peptide; ET-1, endothelin-1GC-MS, gas chromatography-mass spectrometry; 5-HT, 5-hydroxytryptamine; NTG, nitroglycerin; IL-1β, interleukin-1β; NF-κB, nuclear factor kappa B; IκBα, inhibitor of kappa B alpha; NIST, National Institute of Standards and Technology; NO, nitric oxide; PGs, prostaglandins; SD, standard deviation.
